# Prognostic significance of preoperative neutrophil-to-lymphocyte ratio in papillary renal cell carcinoma patients after receiving curative surgery based on a retrospective cohort

**DOI:** 10.1186/s12894-021-00805-8

**Published:** 2021-03-22

**Authors:** Zhilei Zhang, Yongbo Yu, Jilu Zheng, Mingxin Zhang, Haitao Niu

**Affiliations:** 1 Department of Urology, The Affiliated Hospital of Qingdao University, Qingdao, China; 2 Department of Clinical Medicine, Qingdao University, Qingdao, China

**Keywords:** Papillary renal cell carcinoma (PRCC), Neutrophil-to-lymphocyte ratio (NLR), Overall survival (OS)

## Abstract

**Background:**

Inflammatory response biomarkers have been studied as promising prognostic factors in renal cell carcinoma, but few studies have focused on papillary renal cell carcinoma (PRCC). This study was performed to evaluate the prognostic value of the preoperative neutrophil-to-lymphocyte ratio (NLR) in PRCC patients.

**Methods:**

In total, 122 postoperative PRCC patients selected from 366 non-clear cell renal cell carcinoma patients were enrolled from our institution between 2012 and 2020. The optimal cutoff value of the NLR was assessed by receiver operating characteristic (ROC) curve analysis, and the Kaplan–Meier method and Cox's proportional hazards regression models were performed to analyze the association of the NLR with overall survival (OS). In addition, the potential of tumor-node-metastasis (TNM) stage, the NLR and an NLR-TNM system to predict survival were compared with ROC curves, and clinical usefulness of the predicting models were assessed by decision curve analysis.

**Results:**

A threshold value of 2.39 for the NLR for OS analysis was determined by ROC curve analysis. An NLR ≥ 2.39 was associated with a more advanced TNM stage (*P* < 0.01) and larger tumors (*P* < 0.05) than a low NLR, as well as pathological subtype II (*P* < 0.05), and the patients with a high NLR also exhibited significantly worse overall survival outcomes (*P* < 0.05). The NLR was determined to be a significant independent prognostic indicator by univariable and multivariable analyses (HR = 5.56, *P* < 0.05). Furthermore, TNM stage and the NLR were integrated, and the area under the curve (AUC) of for the NLR-TNM system was larger than that of for the TNM system when predicting overall survival (0.84 vs 0.73, *P* = 0.04). Decision curve analysis also demonstrated a better clinical value for the NLR-TNM model to predict the prognosis.

**Conclusion:**

A high preoperative NLR was associated with poor clinical and pathologic parameters in patients with PRCC; moreover, the NLR was also an independent prognostic factor for the OS of patients with PRCC. The NLR-TNM system, which was a model that integrated the NLR with TNM staging, could improve the ability to predict overall survival.

## Background

Renal cell carcinoma (RCC) is a common malignant tumor of the urinary system. In 2018, approximately 175,000 RCC patients died worldwide, and approximately 400,000 new cases were diagnosed [1]. Widespread use of abdominal ultrasound and computed tomography (CT) has increased the number of incidentally detected RCCs [2]. Histologically, RCC can be divided into clear cell renal cell carcinoma (ccRCC), and non-clear cell carcinoma. Papillary renal cell carcinoma (PRCC) is a common subtype of non-clear cell carcinoma, accounting for 15–20% of RCC [3]. The 5-year overall survival rate of PRCC is reported to be significantly higher than that of clear cell carcinoma (80.5% vs 71.3%) [4]. Surgery is still the main treatment of PRCC, and recurrence after surgery often occurs in the advanced stage of PRCC, which may threaten survival and quality of life to a certain extent. Substantial evidence shows that inflammatory cells can induce tumor cell proliferation and metastasis through systemic inflammatory activation; therefore, inflammatory cells may be used to predict tumor prognosis [5–7]. Several indicators of systemic inflammation, such as c-reactive protein (CRP), the neutrophil-to-lymphocyte count ratio (NLR) and the platelet-to-lymphocyte count ratio (PLR), have been studied to predict the prognosis of various tumors [8, 9]. Among these indicators, the NLR is considered a valuable indicator for predicting tumor prognosis, such as that of lung cancer, liver cancer, esophageal cancer and ovarian cancer [10–12]. At present, there are few studies focusing on the prognosis of renal papillary cell carcinoma domestically or globally. Therefore, we used this background to analyze the value of the NLR in evaluating the prognosis of patients with renal papillary cell carcinoma following surgery.

## Methods

### Patients

A total of 366 patients with non-clear cell renal cell carcinoma (nccRCC) who underwent surgical treatment in the department of urology in our hospital from January 2012 to April 2020 were collected.

Inclusion criteria: (1) the medical records were complete. (2) No adjuvant treatment was performed before the operation. (3) The results of blood samplings were obtained within 1 week after admission. (4) The surgical method was partial nephrectomy or radical nephrectomy, and pathological results of specimens were confirmed by more than 2 pathologists as nccRCC with clean cutting edge and no residual.

Exclusion criteria: (1) patients with recent infectious diseases or a history of autoimmune diseases and infectious diseases; (2) patients with a history of primary tumor in other organs; (3) patients with preoperative radiotherapy or chemotherapy; (4) incomplete laboratory and pathological data.

After screening, a total of 122 patients diagnosed as papillary renal cell carcinoma (PRCC) were included in this study. This study was approved by the ethics committee of The Affiliated Hospital of Qingdao University.

### Data collection

During the first week after admission, blood samples were obtained from all the patients. According to the results of peripheral blood test, neutrophil count (× 10^9^/L)/lymphocyte count (× 10^9^/L) was used to calculate the value of NLR. The optimal cutoff value of NLR was identified by an ROC analysis with OS as the primary endpoint, 2.39 was defined as the best cutoff value due to the maximum youden index value (sensitivity: 78.6%; specificity: 70.4%; youden index: 0.49). The cutoff value of CHOL (4.90) were defined by ROC with OS as the endpoint as well.

Follow up strategy: All patients after surgery were considered to be healthy, the first follow-up time in the first 2 years after the operation was every 3 months after operation, for the next 2 years, the follow up time was every 6 months and then annually for the last years, the follow-up included medical history, physical examination, laboratory blood tests and image information, information of death obtained by telephone interview was recorded. The follow-up period was up to 2020-11-01.

### Statistics

Association with NLR between groups was analyzed by Chi-square test and Mann–Whitney U test, and the optimal threshold value of NLR was determined by the receiver operating characteristic (ROC) curve with OS as the primary endpoint. Kaplan–Meier method was utilized to estimate the survival rate of OS, and the survival estimate between groups divided by each cutoff value was compared by log-rank test. Univariable and multivariable COX regression analysis were used to determine the independent prognostic factors of OS. The abilities of different factors to predict OS were compared by the comparisons of the area under curve (AUC) of each parameter, and decision curve analysis was used to assess the clinical usefulness of the predicting models. SPSS ver. 22.0 (SPSS Inc., Chicago, IL, USA), R 4.0.3 (formerly AT&T, now Lucent Technologies), Graphpad Prism 8 (GraphPad Software Inc., La Jolla, CA, USA) and Medcalc v19.1.3 were used to analyze all the statistics. *P* < 0.05 of two sides was considered statistically significant.

## Results

### Patient characteristics

The patient clinicopathological characteristics were listed on Table 1, 122 PRCC patients with 88 (72.1%) males and 34 (27.9%) females aged from 7 to 81 (the mean age was 57.7). According to the histology, these patients were divided into two subtypes 64 (52.5%) I and 58 (47.5%) II, respectively. All patients were underwent curative surgery including radical nephrectomy 64 (52%) and partial nephrectomy 58 (47.5%). Based on the 8th TNM classification of AJCC guideline, 101 (82.7%), 10 (8.2%), 8 (6.6%) and 3 (2.5%) were distributed into I, II, III and IV stage. The median follow up time was 33 months (interquartile range, 16.75–52.5 months), 14 patients were died during the follow-up, and the survival rate of 1 year, 3 years and 5 years were 99.2%, 97.1% and 81.2% in our present study.

**Table 1:** Patient clinical characteristics

Characteristic	No. of patients (%)
Age/years	
< 60	61 (50%)
≥ 60	61 (50%)
Gender	
Male	88 (72.1%)
Female	34 (27.9%)
BMI	
≥ 25	54 (44.3%)
< 25	68 (55.7%)
Diabetes/hyperglycemia, n (%)	
Yes	20 (16.4%)
No	102 (83.6%)
TNM stage (AJCC8th)	
I	101 (82.7%)
II	10 (8.2%)
III	8 (6.6%)
IV	3 (2.5%)
Surgical type	
RN	64 (52.5%)
PN	58 (47.5%)
Tumor size(cm)	
< 7	98 (80.3%)
≥ 7	24 (19.7%)
Subtype, n (%)	
I	64 (55.6%)
II	58 (44.4%)
Serum album(g/l)	
≥ 40	70 (57.4%)
< 40	52 (42.6%)
NLR	
< 2.39	78 (63.9%)
≥ 2.39	44 (36.1%)
CHOL	
< 4.90	65 (53.3%)
≥ 4.90	57 (46.7%)
BUN/Cr	
< 20	87 (57.8%)
≥ 20	35 (42.2%)

*TNM* tumor-node-metastasis, *CHOL* cholesterol, *NLR* neutrophil-to-lymphocyte ratio, *PN* partial nephrectomy, *RN* radical nephrectomy

### Associations between NLR and clinicopathological characteristics of the patients

ROC curve was used to identify the best cut-off value of NLR based on OS (overall survival) as the endpoint. The median preoperative NLR was 1.96 (IQR 1.42–2.82), and the optimal cut-off value of NLR was 2.39 in our study (83.3% sensitivity and 71.8% specificity) by ROC curves analysis, then 78 patients were categorized into the high NLR (≥ 2.39) group and 44 patients were identified into the low NLR (< 2.39) group. Table 2 showed the association between different levels of NLR and clinicopathological characteristics, there were no significant differences between two groups with regard to the age, gender, BMI, surgical type and other clinical characteristics, patients with high NLR tend to have a more advanced TNM stage (*P* < 0.01) and larger tumors (*P* < 0.05) than a low NLR, as well as pathological subtype II (*P* < 0.05).

**Table 2:** Associations between NLR and clinicopathologic characteristics

Variables	NLR < 2.39 (n = 78)	NLR ≥ 2.39 (n = 44)	*P* value
Age/years			0.451
< 60	37 (47.4%)	24 (54.5%)	
≥ 60	41 (52.6%)	20 (45.5%)	
Gender			0.596
Male	55 (70.5%)	33 (75%)	
Female	23 (29.5%)	11 (25%)	
BMI			0.187
≥ 25	38 (48.7%)	16 (36.4%)	
< 25	40 (51.3%)	28 (63.6%)	
Diabetes/hyperglycemia, n (%)			0.363
No	67 (85.9%)	35 (79.5%)	
Yes	11 (14.1%)	9 (250.5%)	
TNM stage (AJCC 8th)			0.001^a^
I	71 (91%)	30 (68.2%)	
II	4 (5.1%)	6 (13.5%)	
III	3 (3.9%)	5 (11.5%)	
IV	0	3 (6.8%)	
Surgical type			0.063
RN	36 (46.2%)	28 (63.6%)	
PN	42 (53.8%)	16 (36.4%)	
Tumor size (cm)			0.011
< 7	68 (87.2%)	30 (68.2%)	
≥ 7	10 (12.8%)	14 (31.8%)	
Subtype, n (%)	0.022		
I	47 (60.3%)	17 (38.6%)	
II	31 (39.7%)	27 (61.4%)	
Serum album (g/l)			0.925
≥ 40	45 (57.7%)	25 (56.8%)	
< 40	33 (42.3%)	19 (43.2%)	
CHOL			0.085
< 4.90	37 (47.4%)	28 (63.6%)	
≥ 4.90	41 (52.6%)	16 (36.4%)	
BUN/Cr			0.274
< 20	53 (67.9%)	34 (77.3%)	
≥ 20	25 (32.1%)	10 (22.7%)	

*TNM* tumor-node-metastasis, *CHOL* cholesterol, *NLR* neutrophil-to-lymphocyte ratio, *PN* partial nephrectomy, *RN* radical nephrectomy

^a^Mann–Whitney U

### Survival analysis

**Fig. 1:**
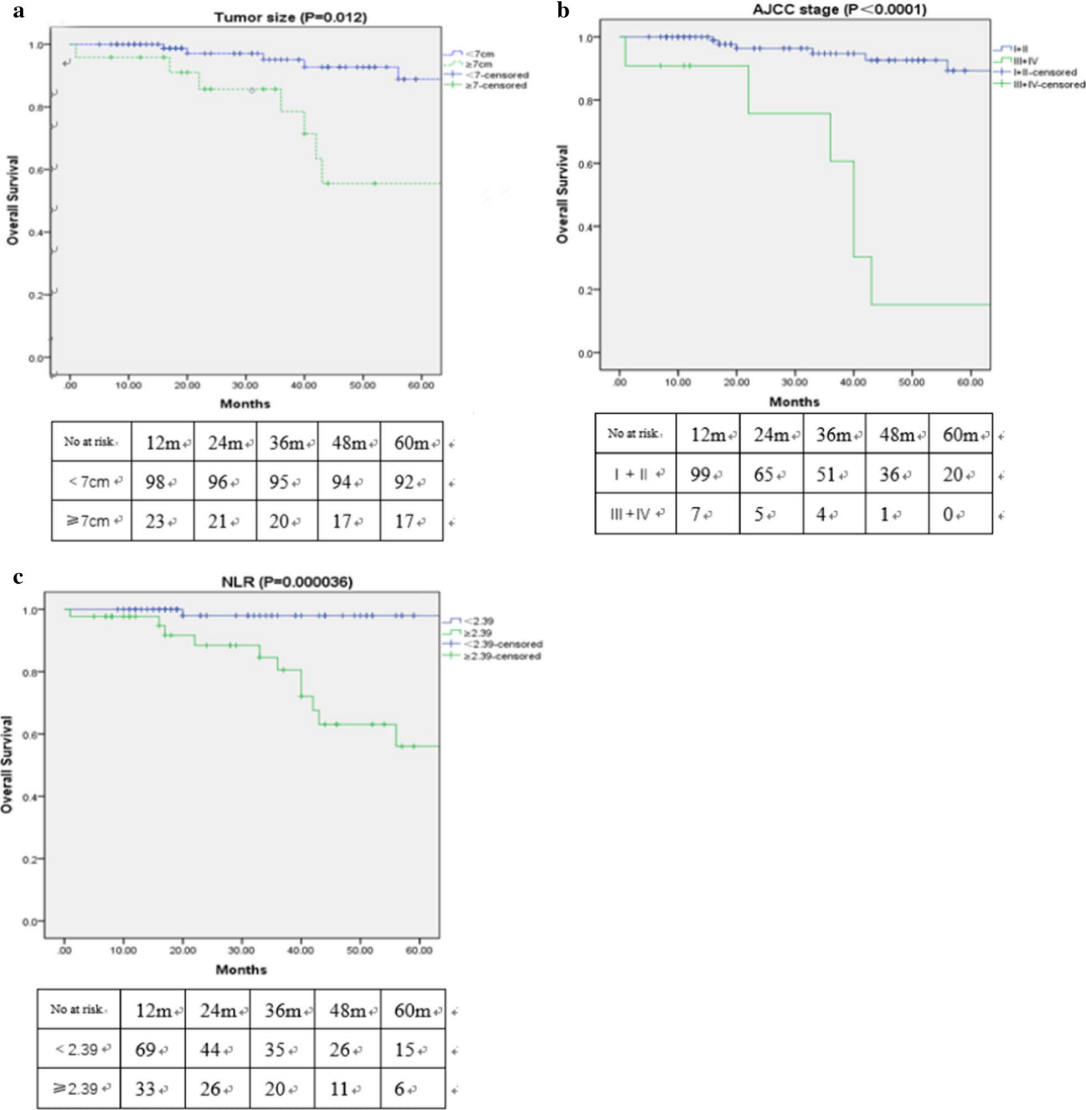
Kaplan–Meier survival curves for OS of patients with PRCC (papillary renal cell carcinoma) according to tumor size (**a**), TNM (AJCC) stage (**b**) and NLR levels (**c**). Patients with larger tumor size, advanced TNM stage and higher NLR were associated with worse OS

The 1 year, 3 year and 5 year survival rates were 100%, 98%and 84%in low NLR group, while the 1 year, 3 year and 5 year survival rates of high NLR group were 97.7%, 80.6% and 56.1%, respectively. The OS time of the high NLR group was shorter than the low NLR group (*P* < 0.01), Fig. 1 showed the Kaplan–Meier survival curves for OS of patients with PRCC according to NLR levels, TNM stage and Tumor size levels, which were all significant predictors for OS (*P* < 0.05) by the univariable analysis (Table 3), Patients with advanced TNM stage and larger tumor size were associated with worse OS.

**Table 3:** Univariable and multivariable analyses of factors in relation to overall survival

Variables	Univariable		Multivariable	
	HR (95% CI)	*P* value	HR (95% CI)	*P* value
Age/years				
≥ 60 versus < 60	1.072 (0.376–3.063)	0.896		
Gender				
(M vs F)	1.149 (0.351–3.7571)	0.818		
BMI				
≥ 25 versus < 25	1.055 (0.365–3.051)	0.921		
Diabetes/hyperglycemia, n (%)				
Yes versus no	1.182 (0.328–4.258)	0.798		
TNM stage (AJCC 8th)				
III + IV versus I + II	12.845 (4.417–37.353)	0.001	4.859 (1.515–15.584)	0.008
Surgical type				
RN versus PN	2.67 (0.744–9.583)	0.132		
Tumor size (cm)				
≥ 7 versus < 7	3.557 (1.24–10.205)	0.018	1.819 (0.60–5.51)	0.290
Subtype, n (%)				
I versus II	0.342 (0.106–1.098)	0.071		
Serum album (g/l)				
< 40 versus ≥ 40	0.641 (0.214–1.92)	0.427		
CHOL				
< 4.9 versus ≥ 4.9	0.988 (0.344–2.838)	0.983		
NLR				
≥ 2.39 versus < 2.39	12.007 (2.675–53.886)	0.001	5.557 (1.059–29.149)	0.043
BUN/Cr				
≥ 20 versus < 20	0.373 (0.082–1.706)	0.203		

*TNM* tumor-node-metastasis, *CHOL* cholesterol, *NLR* neutrophil-to-lymphocyte ratio, *PN* partial nephrectomy, *RN* radical nephrectomy

In the univariable analysis the significant variables (*P* < 0.05) were included into the following multivariate analysis, as shown in Table 3, NLR (HR = 5.56, 95% CI 1.06–29.15, *P* = 0.04) and TNM stage (HR = 4.86, 95% CI 1.52–15.58, *P* = 0.01) was the independent prognostic factors of OS, which demonstrated that patients with a NLR ≥ 2.39 and advanced TNM stage were correlated with a shorter survival time.

### Comparison of the predictive prognostic value of TNM stage, NLR and NLR-TNM model

**Fig. 2:**
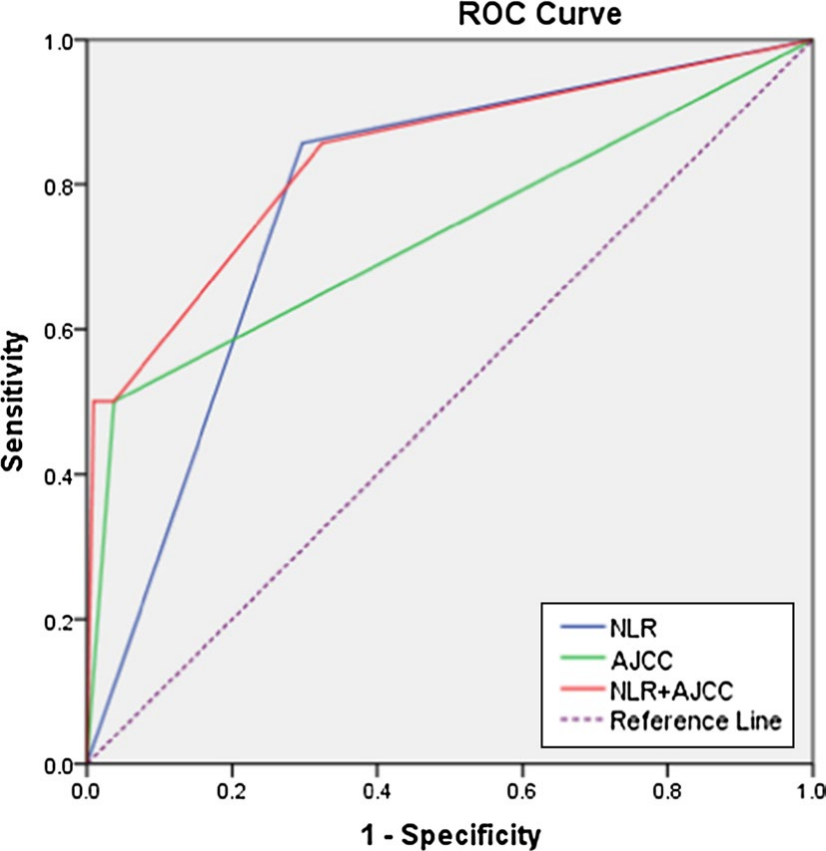
Comparisons of TNM, NLR and NLR-TNM in prognosis prediction of 122 PRCC patients in present study using receiver operating characteristic curves. The AUC of TNM, NLR and NLR-TNM were 0.731 (*P* = 0.005), 0.780 (*P* = 0.001) and 0.839 (*P* < 0.0001), respectively. *TNM* tumor-node-metastasis, *NLR* neutrophil-to-lymphocyte ratio

To evaluate the ability of predicting OS in patients with PRCC, we further drew ROC curves for each parameter to calculated AUC of TNM stage, NLR and NLR-TNM (Fig. 2). The AUC of NLR-TNM system integrated NLR with TNM stage in predicting OS time were relatively higher than that of TNM and NLR alone (0.73 vs 0.78 vs 0.84 for TNM, NLR and NLR-TNM, respectively), which revealed that the predictive ability of NLR was higher than traditional TNM stage, after integrated NLR with TNM stage system, the NLR-TNM showed the better ability in predicting OS time (*P* < 0.05) (Table 4).

**Table 4:** Comparisons of the value of TNM, NLR and NLR-TNM systems in predicting prognosis of overall survival among the patients in present study

Model	AUC (95%CI)	*P*
TNM versus NLR-TNM	0.731 (0.644–0.808) versus 0.839 (0.761–0.899)	0.036
NLR versus NLR-TNM	0.780 (0.696–0.850) versus 0.839 (0.761–0.899)	0.034
NLR versus TNM	0.780 (0.696–0.850) versus 0.731 (0.644–0.808)	0.489

*TNM* tumor-node-metastasis, *NLR* neutrophil-to-lymphocyte ratio

**Fig. 3:**
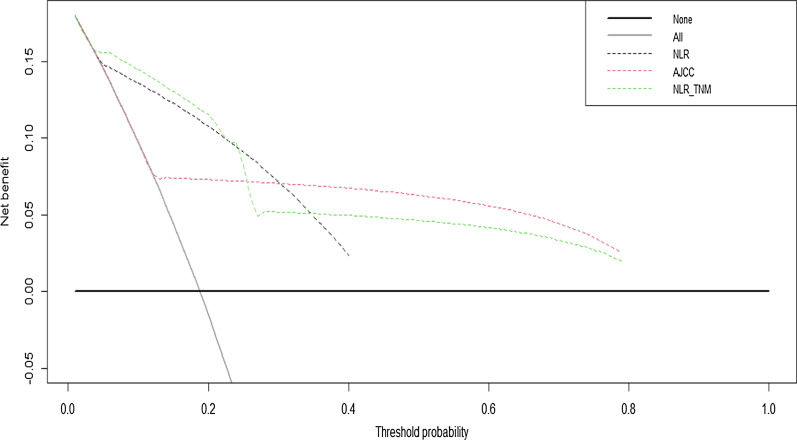
Decision curve analysis for the TNM, NLR and NLR-TNM model. Notes: The y-axis measures the net benefit. The dotted line represents the model of TNM, NLR and NLR-TNM. The thin solid line represents the assumption that all patients are intervened. Thin thick solid line represents the assumption that no patients are intervened. The decision curve of NLR-TNM model showed the most benefit than the other two models

Figure 3 showed the decision curves of the predicted models of NLR, TNM stage and NLR-TNM, and a positive net benefit was compared among them, which demonstrated that using this NLR-TNM model in the current study to predict OS added more benefits than the other two models.

## Discussion

In our study, we respectively analyzed 122 PRCC patients who underwent curative surgical treatment. An NLR ≥ 2.39 was closely correlated with a relatively advanced pathological stage and large tumor volume. In addition, the results demonstrated that a high preoperative NLR was an independent prognostic factor associated with shortened OS. These results were in line with other previous studies [13–15], which implies that the NLR is relevant for predicting poor clinical characteristics. Furthermore, the NLR-TNM system was relatively more accurate in predicting overall survival than the TNM stage or NLR alone.

Several immunohistochemical biomarkers and nomograms have been confirmed to be independent prognostic factors for the survival of RCC patients [16, 17]. The NLR is a tumor marker easily obtained from the peripheral blood. Previous research has reported that the NLR has been used to evaluate the tumor prognosis of localized renal cell carcinoma [18, 19], but most studies have focused on clear cell renal cell carcinoma, and there is a lack of studies on predictive models for pathological subtypes of papillary renal cell carcinoma. Huang et al. first reported that the NLR was associated with the RFS (recurrence-free survival) of non-metastatic papillary cell carcinoma patients [20], which revealed that an NLR≥3.6 was an independent predictor of RFS. This was similar to the conclusion of our study. Most different cutoff levels for the NLR with prognostic value for RCC range from 3 to 5 [21], which was not consistent with our study, Most of the patients with PRCC in our study were staged early, and PRCC is a tumor with relatively low malignancy, which may contribute to this difference. Our study also found that a high preoperative NLR was related to poor overall survival in PRCC, indicating that the NLR could be used as an independent prognostic factor to predict prognosis.

The inflammatory response is an important factor in the occurrence of malignant tumors, and tumor cells release a large number of inflammatory factors to change the tumor microenvironment, which can help tumor cells escape the host immune response. This escape leads to the proliferation and distant metastasis of tumor cells [22] and promotes the transformation of normal cells into tumor cells. Recent studies have shown that neutrophils can produce and release active cellular factors, such as IL6-1, IL-6 and VEGF, which changes the balance of inflammatory and anti-inflammatory in the tumor microenvironment [23]. Lymphocytes are important immune cells that contribute to tumor immune surveillance, which can inhibit tumor invasion. The NLR is a more accurate indicator of the inflammatory response level than either single factor composing the NLR. Therefore, a large number of studies have shown that a high NLR is associated with an advanced stage and a poor prognosis [24, 25], which was also supported by our study.

Since 2015, targeted therapy and immune checkpoint inhibitors have gradually become the main treatments for advanced metastatic renal carcinoma [26]. Vascular endothelial growth factor (VEGF) and vascular endothelial growth factor receptor (VEGFR) have become crucial targets of anticancer therapy because of their antiangiogenic functions. VEGF is a cellular activity factor produced by neutrophils, and whether the NLR can be used as a potential marker for the effect of targeted therapy remains to be determined. One study showed that the NLR was reduced by adjuvant sunitinib therapy for 4 weeks, with one endpoint of RFS improved [27]. Moreover, the NLR has been reported to be lower after the immunotherapy in metastatic RCC, with improved survival outcomes [28]. The systematic inflammatory response will be a very interesting focus of further research after neoadjuvant or adjuvant therapy in advanced RCC and PRCC.

Our study also had several limitations. First, our study was a respective study with a single-center, and the low incidence of PRCC led to the small sample of patients, so we did not divide the patients into metastatic PRCC and non-metastatic PRCC. Second, we did not evaluate the NLR after surgery, which limits the value for predicting prognosis. Third, due to a small number of disease specific deaths, we analyzed only OS as the primary endpoint.

## Conclusion

This study showed that high preoperative NLR was associated with poor clinical and pathologic parameters of patients with PRCC. Moreover, NLR was also an independent prognostic factor of OS for patients with PRCC. The model of NLR-TNM integrating NLR with TNM stage could improve the ability of predicting overall survival.

## Data Availability

Records and data pertaining to this study are in the patient’s secure medical records in the Affiliated Hospital of Qingdao University, and the records and data could be accessed from ZZ.

## Abbreviations

PRCC: Papillary renal cell carcinoma
NLR: Neutrophil-to-lymphocyte ratio
TNM: Tumor-node-metastasis
ROC: Receiver operating curve
